# *Odoribacter splanchnicus*-derived extracellular vesicles alleviate inflammatory bowel disease by modulating gastrointestinal inflammation and intestinal barrier function via the NLRP3 inflammasome suppression

**DOI:** 10.1186/s10020-025-01063-2

**Published:** 2025-02-11

**Authors:** Jinfu Zhuang, Zhicheng Zhuang, Bin Chen, Yuanfeng Yang, Hengkai Chen, Guoxian Guan

**Affiliations:** Department of Colorectal Surgery, The First Affiliated Hospital of Fujian Medical University, 20th, Chazhong Road, Fuzhou, Fujian Province 350005 P. R. China

**Keywords:** Inflammatory bowel disease, *Odoribacter splanchnicus*, Extracellular vesicles, Intestinal tight junction, NLRP3 inflammasome

## Abstract

**Background:**

Extracellular vesicles (EVs) derived from specific bacteria exert therapeutic potential on inflammatory diseases. Previous reports suggest the protective role of *Odoribacter splanchnicus* (*O.splanchnicus*) in inflammatory bowel disease (IBD). The effect of EVs derived from *O.splanchnicus* (Os-EVs) and the underlying mechanism on IBD were surveyed here.

**Methods:**

Os-EVs were derived with ultracentrifugation before characterization by transmission electron microscopy and nanoparticle tracking analysis. Based on IBD model mice induced by dextran sulfate sodium (DSS), the effects of Os-EVs on IBD symptoms, intestinal barrier dysfunction, and colonic apoptosis, inflammation as well as NLRP3 inflammasome activation were analyzed. NLRP3 knockout mice were exploited to judge the role of NLRP3 in Os-EVs against IBD.

**Results:**

Os-EVs were typically shaped as a double concave disc (average diameter = 95 nm). The administration of Os-EVs attenuated DSS-induced body weight loss, colon shortening, disease activity index score, and histological injury in mice. Os-EVs could also relieve intestinal barrier dysfunction and colonic apoptosis, as evidenced by the up-regulation of zona occludens-1 and Occludin and the decrease of TUNEL-positive staining in colonic tissues of IBD mice. Os-EVs downregulated the expression of the interleukin-1β (IL-1β), tumor necrosis factor-α, and IL-6, and elevated IL-10, accompanied by blockage of the NLRP3 inflammasome activation in DSS-induced mice. Furthermore, NLRP3 knockout mice experiments revealed that the protective role of Os-EVs in IBD relies on regulating NLRP3.

**Conclusion:**

Our finding indicated that Os-EVs effectively ameliorated IBD through repressing NLRP3, strongly supporting the potential of probiotic-derived EVs for alleviating IBD.

## Background

As a global health problem, inflammatory bowel disease (IBD) is a heterogeneous and chronic autoimmune disease with the characterization of chronic recurrent intestinal inflammation (Liu and Stappenbeck 2016). It is widely accepted that IBD is attributed to many factors, which include environmental factors, genetic predispositions, excessive immune response, and so on (de Souza and Fiocchi 2016; Lees et al. 2011). Even though the pathophysiology of IBD remains insufficiently understood, a consensus of evidence supports that IBD is closely associated with dysbiosis of gut microbiota (Ni et al. 2017). For example, an increasing number of metagenomic studies on IBD patients uncovered widespread gut microbiota disturbances (Ekstedt et al. 2024; Sartor and Wu 2017), which the loss of probiotics may explain. In the last few decades, probiotic supplementation with an attempt to improve intestinal dysbiosis has emerged as a hopeful strategy against gastrointestinal disorders (Schieber et al. 2015; Lemon et al. 2012). Various research revealed that probiotics derived from healthy individuals effectively restore gut microbiota structure, govern immunity, and protect the intestinal barrier (Smillie et al. 2018).

Extracellular vesicles (EVs) are lipid bilayer-enclosed globular particles with an average diameter of ranges from 20 to 500 nm, which play important roles in cellular communication (Shen et al. 2022). EVs are becoming a hotspot in biomedical research field because of their full of parental functional molecules (Liu et al. 2018; Kumar et al. 2024). Growing evidence suggests that EVs derived from bacteria, especially probiotics, can favorably govern host immune response, protect intestinal barrier integrity, and exhibit additional beneficial effects (Champagne-Jorgensen et al. 2021; Kim et al. 2016). Notably, when administration, EVs may not cause injurious effects such as bacteremia since they are not bacteria themselves (Liu et al. 2018). Recently, a research demonstrated that *Akkermansia muciniphila*-derived EVs alleviate colitis by modulating intestinal homeostasis (Wang et al. 2023). Besides, EVs derived from *Lactobacillus johnsonii* were also demonstrated to govern intestinal barrier homeostasis through promoting the M2 polarization of macrophages in diarrheic disease (Tao et al. 2024). Even though the specific molecular mechanisms have not been completely elucidated, probiotic EVs provide novel insight as a promising strategy to struggle intestinal inflammatory disorders.

In comparison with healthy controls, a decrease of intestinal short-chain fatty acid (SCFA) content together with a lower abundance of the dominant bacterial producers were linked with active IBD (Parada Venegas et al. 2019). *Odoribacter splanchnicus* (*O. splanchnicus*) is an SCFA-producing microbiota belonging to the order Bacteroidales (Göker et al. 2011). Research has shown that a decreased abundance of *O. splanchnicus* is associated with the development of different microbiota-associated diseases, which included IBD (Wang et al. 2018; Lewis et al. 2015). For example, Li et al. recently revealed the reduced abundance of *O. splanchnicus* in patients with ulcerative colitis (Li et al. 2021), which is consistent with that previously reported in patients with Crohn’s disease (Morgan et al. 2012). Additionally, a previous study revealed that *O. splanchnicus* as an immune-reactive bacterial core strain is related to the clinical response of colitis patients during fecal microbiota transplantation therapy (Lima et al. 2022). Moreover, *O. splanchnicus* intervention effectively alleviates colitis by a mechanism of neutrophil extracellular traps formation in mice (Xu et al. 2023). Considering the anti-inflammatory functions of *O. splanchnicus* and its possible application for treating intestinal inflammatory diseases, it is of close attention to look into the potential of EVs secreted by *O.splanchnicus* (Os-EVs) in treating IBD.

By using specified pathogen-free (SPF) mice stimulated with dextran sulfate sodium (DSS) and interventions with Os-EVs, the present study revealed the significant protection of Os-EVs against DSS-induced IBD and preliminarily uncovered the underlying mechanism, suggesting a novel strategy for preventing and treating IBD.

## Methods

### Bacterial EVs isolation and characterization

The *O.splanchnicus* strain (NCTC 10825 [1651/6]) obtained by the American Type Culture Collection (ATCC) was anaerobically grown in the Gifu anaerobic medium with vigorous shaking at 37 °C.

The bacterial-cultured supernatant was harvested and subsequently purified with centrifugation and a 0.22-µm membrane filter. Briefly, the harvested supernatant was centrifuged for 10 min at 1500 × g, transferred to new tubes, and centrifuged for 30 min at 15,000 × g. Subsequently, the supernatant was filtered to remove bacterial debris (0.45 μm) and contaminated proteins (0.22 μm). Then, in order to isolate Os-EVs, the purified bacterial-cultured media was subjected to an hour of 200,000 × g ultracentrifugation. All centrifugations were performed at 4 °C. Next, to obtain pure Os-EVs, the pellets were collected to resuspend with phosphate-buffered saline (PBS) and filtered by a 0.22-µm syringe filter for quality control following the guidelines suggested by the International Society of Extracellular Vesicles (ISEV) (Welsh et al. 2024). Finally, the protein concentration of the pure Os-EVs was quantified with a BCA assay kit.

Transmission electron microscope (TEM) and nanoparticle tracking analysis (NTA) with Nano-ZS 90 dynamic light scattering were applied to observe the morphology, number, and size distribution of Os-EVs. The remaining Os-EVs were stored at -20 °C for further analyses.

### Experimental animals and establishment of DSS-induced IBD model

In this study, all experimental processes regarding animals were performed following the Administration of Laboratory Animals promulgated by the Science and Technology Commission of the People’s Republic of China. The study was approved by the Animal Care and Use Committee of the First Affiliated Hospital of Fujian Medical University.

Wild-type (WT) C57BL/6 and NLRP3 knockout (NLRP3-/-) mice (male, 6–8 weeks) purchased from Laboratory Animal Center of Fujian Medical University, Inc. were housed under SPF laboratory with the temperature maintained at 24 ± 1 °C and provided free access to food and water. Before experiments, all mice were subjected to 7-day acclimatization. Then, both the WT and NLRP3-/- mice were randomly divided into three subgroups: the control, DSS, and DSS + Os-EVs groups. To induce IBD in mice, 3% (w/v) DSS was administrated in drinking water for 7 days, whereas mice of the control group received water without DSS. In the first 5 days, the mice in the DSS + Os-EVs group received oral administration of Os-EVs (200 µg per mouse, suspended in 200 µL of sterile PBS) once every day, while the control and DSS groups received the same volume of sterile PBS (Fig. 2A).

To evaluate the severity of IBD, the weight loss as well as disease activity index (DAI) of each mouse were monitored every day. DAI scores were determined on the basis of the degree of weight loss, diarrhea, and hematochezia status, as previously reported by Sann and colleagues (Sann et al. 2013). Briefly, weight loss was scored as 0 to 4, weight loss that less than 2% was scored as 0, that ranged from 2 to 5% was scored as 1, that ranged from 5 to 10% was scored as 2, that ranged from 10 to 15% was scored as 3, or that more than 15% was scored as 4; normal stool was scored as 0, the softer stool was scored as 1, moderate diarrhea was scored as 2, or diarrhea was scored as 3; and bleeding was scored as 0 to 3, 0 means no rectal bleeding, 1 represents weak hemoccult, 2 means that there was blood in stool, or 3 means that fresh rectal bleeding was observed. On the last day, all mice were anesthetized by intraperitoneally injecting with 1% sodium pentobarbital followed by being euthanized with cervical dislocation. Finally, the entire colon was removed and harvested for measuring colon length and the subsequent analyses.

### Histological analysis

After being separated and cut longitudinally, colons from each group were subjected to 4% paraformaldehyde fixation, gradient dehydration, embedding in paraffin, and subsequently sectioned as 5 μm slices. Then, the Hematoxylin & Eosin (H&E) staining kit (Beyotime) was exploited for histopathological staining on microscopic slides as per the manufacturer’s protocol. Finally, the morphological images were obtained using the BX43 microscope (Olympus, Japan), and the scores of histopathological change for colon lesions were carried out according to the scoring criteria (Wirtz et al. 2017), which ranged from 0 (no change) to 6 (widespread tissue damage and extensive cellular infiltration).

### Immunofluorescent (IF) localization of tight junction protein

The expression and distribution of the Zonula occludens-1 (ZO-1) in colonic slices were analyzed by IF staining based on the procedures described in the previous study (Kuo et al. 2021). After dewaxing and hydration, antigen retrieval, and blocking procedures, colonic slices were subjected to incubation with the primary antibodies against ZO-1 (Beyotime, AF8394) overnight at 4 °C followed by incubation with the secondary antibody (Beyotime, A0208) for 30 min. Finally, the colonic slices were rinsed with PBS prior to staining with 4,6′-Diamidino-2-phenylindole (DAPI), subsequently observed under the BX43 microscope.

### Western blot (WB) analysis

Colonic samples were lysed with RIPA Lysis Buffer (Solarbio) prior to the determination of protein concentration with the BCA Protein Assay Kit (#PC0020, Solarbio). Next, the equal amount of protein samples (20 µg per lane) were subjected to SDS polyacrylamide gel separation, followed by being transferred onto the 0.45 μm polyvinylidene fluoride membranes. Then, blockage of membranes lasted 1 h, followed by incubating membranes with primary antibodies against ZO-1, Occludin (Beyotime, AF7644), NLRP3 (Beyotime, AF2155), pro-caspase-1 (Abcam, ab138483), cleaved-caspase-1 (ThermoFisher, PA5-99390), apoptosis-associated speck-like protein containing a CARD (ASC) (Beyotime, AF6234), and β-actin (Beyotime, AF0208) (served as an internal control) at 4 °C overnight. Finally, after incubation with the appropriate HRP-coupled secondary antibody, signals of the studied proteins were visualized by chemiluminescence using an immobilon western HRP substrate (Millipore). Analysis of the data was finally performed using ImageJ software V1.53 (NIH).

### Terminal deoxynucleotidyl transferase dUTP nick end labeling (TUNEL) staining

The apoptosis in IBD mice’s colon tissue was investigated with TUNEL staining based on the In-Situ Cell Death Detection Kit. The nucleus was stained with DAPI while the apoptotic cell was stained with TUNEL. Under the BX43 microscope, TUNEL-positive cells were counted (at least 10 fields).

### Quantitative real-time polymerase chain reaction (RT-qPCR)

TRIzol reagent (Thermo, Rockford, IL, USA) was applied to extract total RNA from colonic samples. After reverse transcription was completed with the PrimeScript RT reagent kit with genomic DNA Eraser (Takara), the complementary DNA was subjected to qPCR analysis using SYBR Premix Ex Taq II (Takara). Primer sequences are shown as follows: IL-1β, forward GGGGCGTCCTTCATATGTGT, reverse GGCAGCTCCTGTCTTGTAGG; IL-6, forward GAGGTGAGTGCTTCCCCATC, reverse TTGCATCTGGCTTTGTTCGC; TNF-α, forward ACTGATGAGAGGGAGGCCAT, reverse CCGTGGGTTGGACAGATGAA; IL-10, forward GCAAGGGTGTCTCCTTCCTC, reverse CTTGTTACACTCGCCCCCTT; β-actin, forward TCCTATGGGAGAACGGCAGA, reverse TCCTTTGTCCCCTGAGCTTG. Relative gene expression values were determined by the 2^−ΔΔCT^ method, based on the normalization of the housekeeping β-actin.

### Enzyme-linked immunosorbent assay (ELISA)

The serum levels of IL-6, TNF-α, IL-1β, and IL-10 were determined following the instructions for the Mouse IL-6 ELISA kit (Solarbio, SEKM-0007), Mouse TNFα High Sensitivity ELISA Kit (ThermoFisher, BMS607-2HS), Mouse IL-1 beta ELISA Kit (ThermoFisher, BMS6002), and Mouse IL-10 Immunoassay (Bio-Techne, M1000B-1).

### Immunohistochemical (IHC) analysis

After the elimination of the endogenous peroxidase activity and antigen reparation, the primary antibodies against NLRP3 and Caspase 1 p20 were used to incubate with 5-µm paraffin-embedded colonic sections overnight at 4 °C. Subsequently, the sections were subjected to incubation with the secondary antibodies for 30 min and visualization by 3, 3-diaminobenzidine for 10 min. Images were taken at 20× the original magnification.

### Statistical analysis

GraphPad Prism version 8.1.0 was applied to conduct the statistical analyses. Comparative analyses were carried out using one-way analysis of variance. Evaluation of *P* < 0.05 was accepted as statistically significant.

## Results

### Os-EVs attenuate DSS-induced IBD in mice

**Fig. 1:**
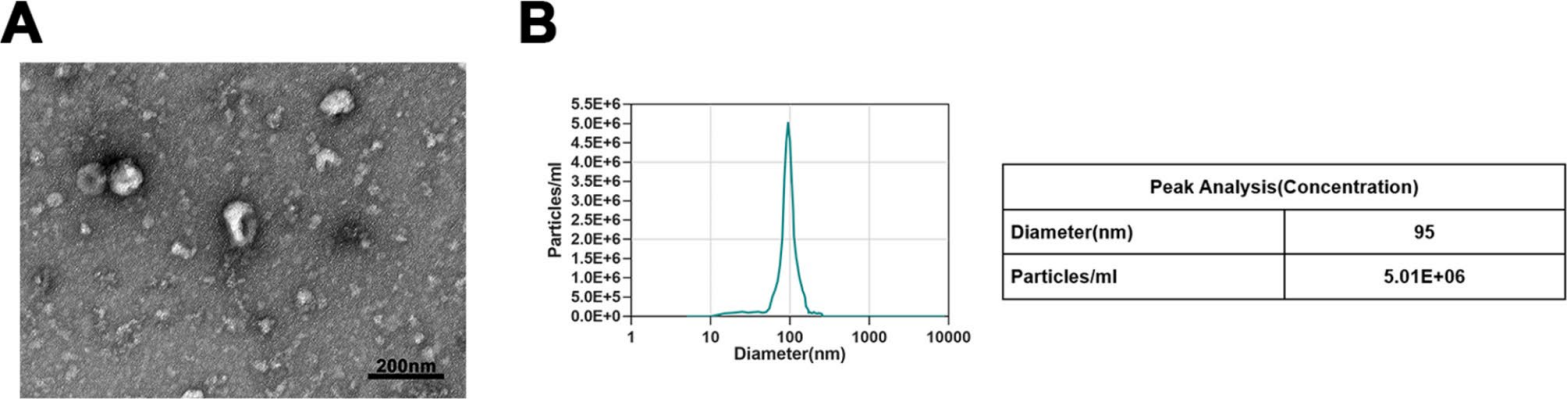
Identification and characterization of Os-EVs. To isolate and purify Os-EVs, the purified bacterial-cultured media was subjected to an hour of 200,000 × g-force ultracentrifugation, followed by resuspend with PBS and filtered by a 0.22-µm syringe filter. **A**. TEM was utilized to capture the images of the isolated Os-EVs (Scale bars represent 200 μm). **B**. NTA was exploited to analyze the size distribution of the isolated Os-EVs

Initially, Os-EVs were derived from *O.splanchnicus* by using ultracentrifugation and characterized by TEM and NTA. The characterization showed the Os-EVs typically shaped as a double concave disc, of which the average diameter is 95 nm (Fig. 1A and B).

**Fig. 2:**
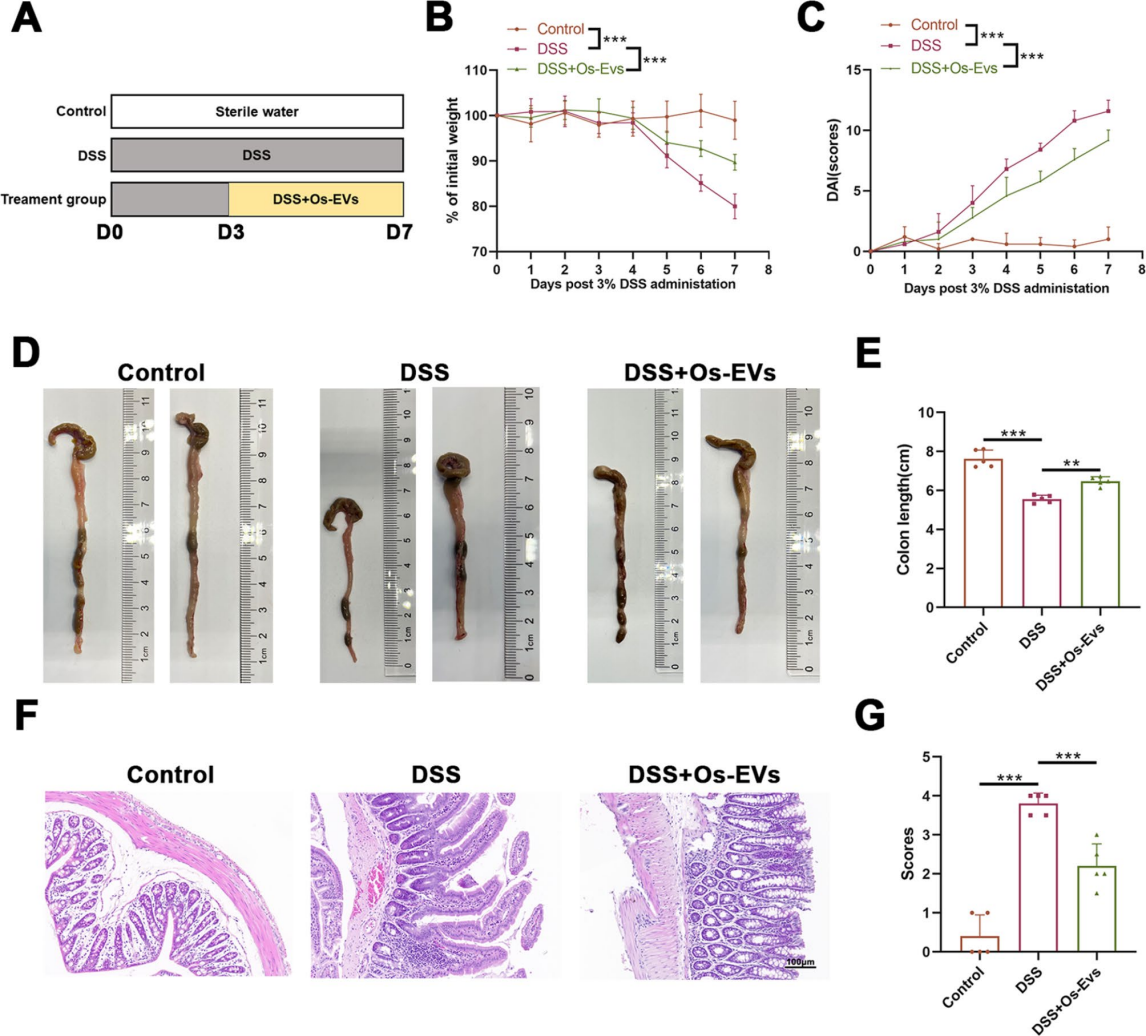
Os-EVs attenuate DSS-induced IBD in mice. **A**. Flow diagram illustrating the experiment schedule on mice: mice were randomly divided into three subgroups: the control, DSS, and DSS + Os-EVs groups. To induce IBD in mice, 3% (w/v) DSS was administrated in drinking water for 7 days, whereas mice of the control group received water without DSS. In the first 5 days, the mice in the DSS + Os-EVs group received oral administration of Os-EVs once every day, while the control and DSS groups received the same volume of sterile PBS. **B**. Changes in body weight of each mouse were monitored every day (*n* = 5). **C**. DAI scores of each mouse were determined on the basis of the degree of weight loss, diarrhea, and hematochezia status after the administration of 3% DSS (*n* = 5). **D**. Representative images of the colon from the control, DSS, and DSS + Os-EVs groups (*n* = 2). **E**. Length of the harvested colon from three groups (*n* = 5). **F**. Representative H&E-stained colonic slices among three groups (Scale bars represent 100 μm.). **G**. Histopathological scores were determined based on H&E-stained colonic slices among three groups (*n* = 5). Note: ** means *P* < 0.01 and *** means *P* < 0.001

Then, to explore the protection of Os-EVs on DSS-stimulated IBD, Os-EVs were utilized to treat mice for the first 5 days during the administration of DSS (Fig. 2A). Results demonstrated that Os-EVs pretreatment effectively prevented the DSS-induced body weight loss, bloody diarrhea, as well as the increased DAI scores in mice (Fig. 2B and C). Besides, Os-EVs pretreatment also alleviated the colon length shortening (Fig. 2D and E). Histological assessment based on colon H&E stain indicated that the DSS-induced histopathological injuries such as inflammatory cell infiltration and intestinal mucosal destroy were obviously attenuated in IBD mice treated with Os-EVs (Fig. 2F), as evidenced by histopathology scores (Fig. 2G). These outcomes support that Os-EVs significantly ameliorated the symptoms of IBD in DSS-induced mice.

### Os-EVs prevent against DSS-induced intestinal mucosal barrier dysfunction in IBD mice

**Fig. 3:**
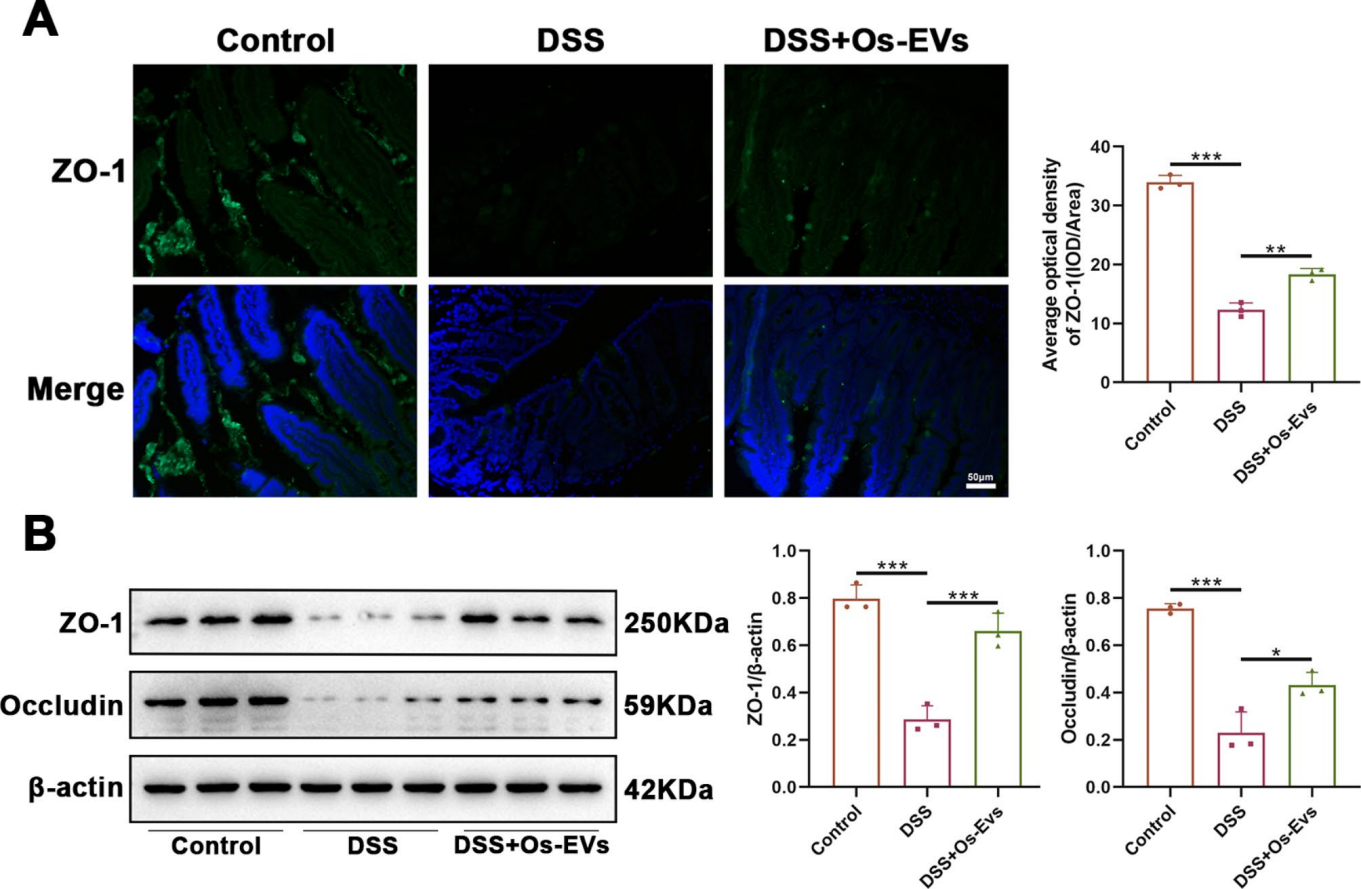
Os-EVs prevent against DSS-induced intestinal mucosal barrier dysfunction in IBD mice. **A**. Immunofluorescent staining of ZO-1 in the colonic slices from each group (Scale bars represent 50 μm) (*n* = 3). **B**. Protein levels of ZO-1 and Occludin in colon tissue analyzed by WB (*n* = 3). Note: *means *P* < 0.05, ** means *P* < 0.01 and *** means *P* < 0.001

It is acknowledged that tight junction proteins, such as ZO-1 and Occludin, are critical components of the intestinal mucosal barrier, which play a critical function on colonic homeostasis by mediating robust mechanical stability (Landy et al. 2016). Hence, IF staining of ZO-1 was histologically performed on colonic sections to determine the role of Os-EVs in intestinal mucosal integrity. As depicted in Fig. 3A, the colonic slices of the control mice displayed a solid connection structure; while the epithelial tight junction proteins became blurred and loose in the IBD mice, which supported DSS-induced intestinal barrier dysregulation in mice. Notably, compared with the DSS group, higher levels of ZO-1 expression in the colonic slices were observed in the DSS + Os-EVs group (Fig. 3A), suggesting that pretreatment with Os-EVs could partially rebuild intestinal tight junction connections. Furthermore, the expression of ZO-1 and Occludin in colon tissue detected by WB confirmed this result. WB analysis showed a markedly decreased expression of ZO-1 and Occludin was observed in the colon tissue of the DSS group when in comparison to the control group, while Os-EVs pretreatment could increase these protein levels in the colon tissue of DSS-induced IBD mice (Fig. 3B). Collectively, our results demonstrated that Os-EVs pretreatment safeguarded intestinal barrier function during DSS-induced IBD.

### Os-EVs alleviate intestinal epithelial cell apoptosis and colonic inflammation in DSS-induced IBD mice

**Fig. 4:**
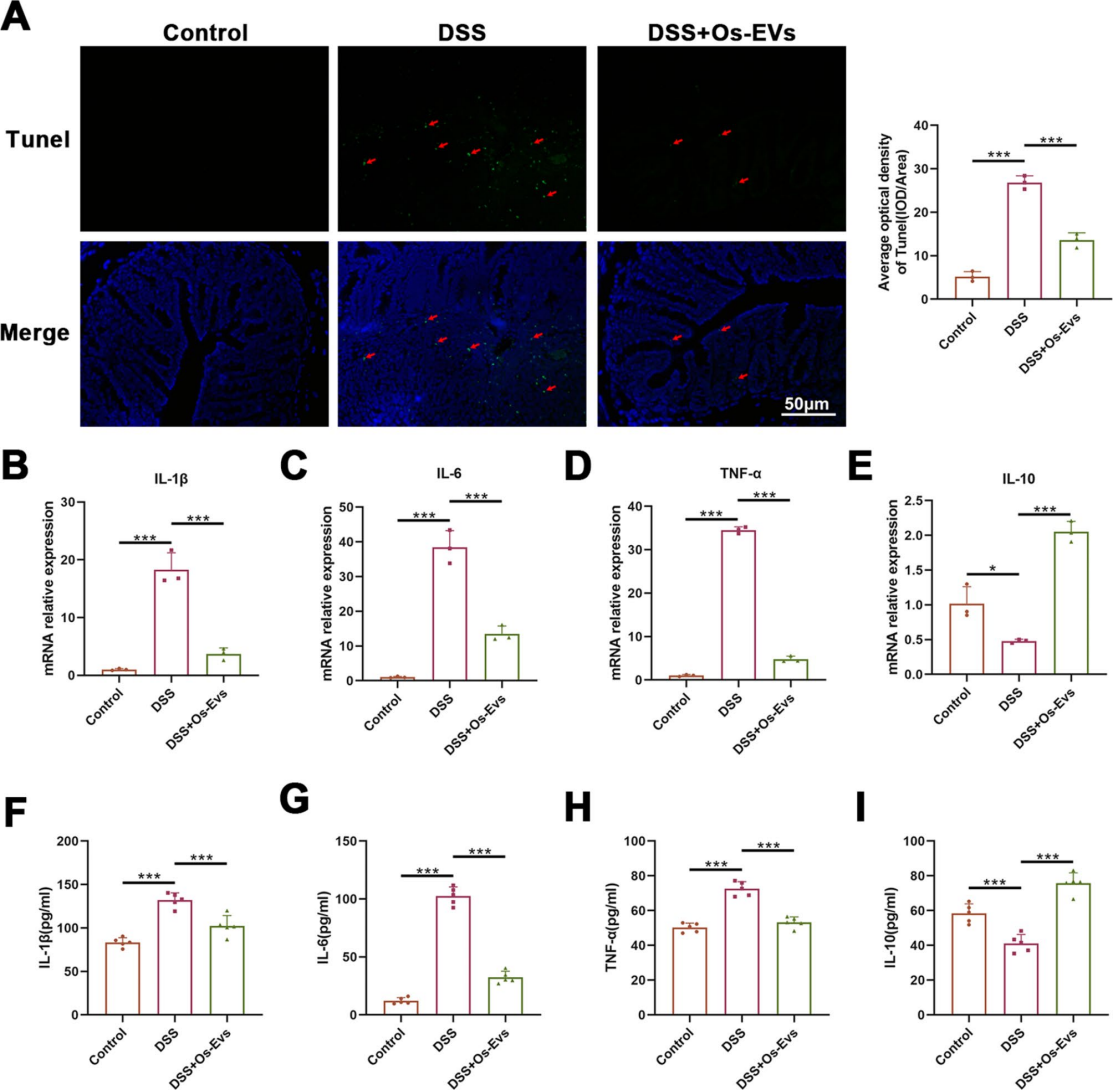
Os-EVs alleviate colon apoptosis and inflammation in DSS-induced IBD mice. **A**. TUNEL staining for colonic section (*n* = 3). **B**-**E**. The mRNA expression of IL-1β, IL-6, TNF-α, and IL-10 in colon tissue (normalized to β-actin) (*n* = 3). **F**-**I**. Serum IL-6, IL-1β, TNF-α and IL-10 levels were measured by ELISA from the mice (*n* = 5). Note: *means *P* < 0.05 and *** means *P* < 0.001

The intestinal barrier dysfunction in IBD is also attributed to intestinal epithelial cell apoptosis, the present study, therefore, assessed the apoptosis in the colonic tissue with TUNEL staining. The result demonstrated a reduced number of apoptotic intestinal epithelial cells with green fluorescence (red arrows) in the colonic slices of the DSS + Os-EVs group compared to those of the DSS mice without treatment (Fig. 4A), suggesting that Os-EVs may prevent intestinal barrier dysfunction by reducing colonic intestinal epithelial cell apoptosis.

Due to the pivotal role of cytokines in IBD pathogenesis (Neurath 2014), the transcription levels of inflammation-related cytokines in the colon tissues were analyzed by RT-qPCR. As expected, significant increases in the mRNA levels of IL-1β (Fig. 4B), IL-6 (Fig. 4C), as well as TNF-α (Fig. 4D**)**, and decreased levels of IL-10 mRNA (Fig. 4E) were detected in the colonic tissue of DSS-induced mice as compared with the control mice, while the pretreatment of Os-EVs could reduce the mRNA expression of these proinflammatory cytokines and improve anti-inflammatory cytokine expression. The anti-inflammatory effect of Os-EVs on DSS-induced mice was further confirmed by detecting serum levels of these cytokines. Results from ELISA indicated there was elevated secretion of IL-1β (Fig. 4F), IL-6 (Fig. 4G), as well as TNF-α (Fig. 4H), and reduced secretion of IL-10 (Fig. 4I) in DSS-induced mice. Notably, pretreatment with Os-EVs reversed this trend by reducing secretion of IL-1β, IL-6, as well as TNF-α, and enhancing IL-10 production, further supporting that Os-EVs have an anti-inflammatory repair effect on IBD in DSS-induced mice.

### Os-EVs prohibit the activation of NLRP3 inflammasome in DSS-induced IBD mice

**Fig. 5:**
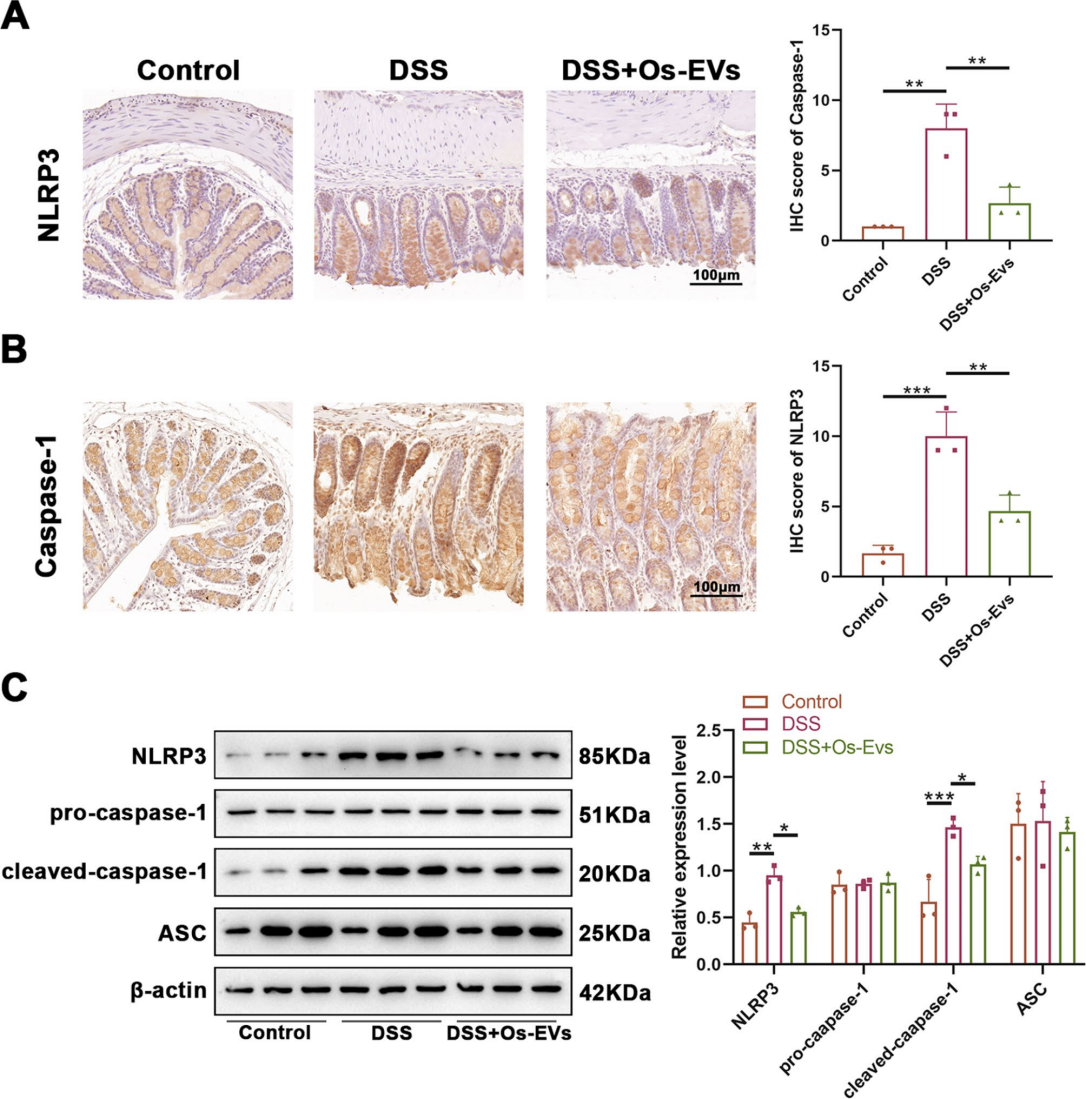
Os-EVs inhibit the activation of NLRP3 inflammasome in DSS-induced IBD mice. **A**. IHC analysis for colonic NLRP3 expression (Left-representative images of colon sections; right-IHC scores (*n* = 3)). **B**. IHC analysis for colonic Caspase-1 expression (Left-representative images of colon sections; right-IHC scores (*n* = 3)). **C**. Expression levels of NLRP3 signaling-related proteins (NLRP3, ASC, pro-caspase-1, and cleaved-caspase-1) in colon tissue analyzed by WB normalized to β-actin (*n* = 3). Note: *means *P* < 0.05, ** means *P* < 0.01 and *** means *P* < 0.001

Considering that the high level of IL-1β in DSS-induced IBD is closely related to the NLRP3 inflammasome signaling pathway (Mai et al. 2019). To observe whether the suppression of IL-1β by Os-EVs is due to the blockage of NLRP3 signaling, IHC analysis was conducted to observe colonic NLRP3 and caspase-1 p20 expression. It has been found high expression of caspase-1 p20 and NLRP3 in the colon of the DSS-induced mice (Fig. 5A and B), indicating the NLRP3 inflammasome activation. In contrast, pretreating with Os-EVs could reduce DSS-induced up-regulation of these two proteins (Fig. 5A and B), which hinted at the suppression of the NLRP3 inflammasome by Os-EVs. WB analysis was used to further confirm the NLRP3 inflammasome activation in the DSS-induced colonic epithelium of IBD mice compared to the suppression in IBD mice pretreated with Os-EVs. Consistent with our IHC data, DSS-induced up-regulation of NLRP3 and cleaved caspase-1 in mice was decreased by the pretreatment of Os-EVs (Fig. 5C). Moreover, ASC, the adaptor of NLRP3 inflammasome, of which expression was enhanced by DSS, was also remarkably repressed by Os-EVs (Fig. 5C). The results presented above indicated that the safeguarding impact of Os-EVs on DSS-induced IBD could be associated with NLRP3 inflammasome suppression.

### The protection of Os-EVs for DSS-induced IBD was diminished in NLRP3-deficient mice

**Fig. 6:**
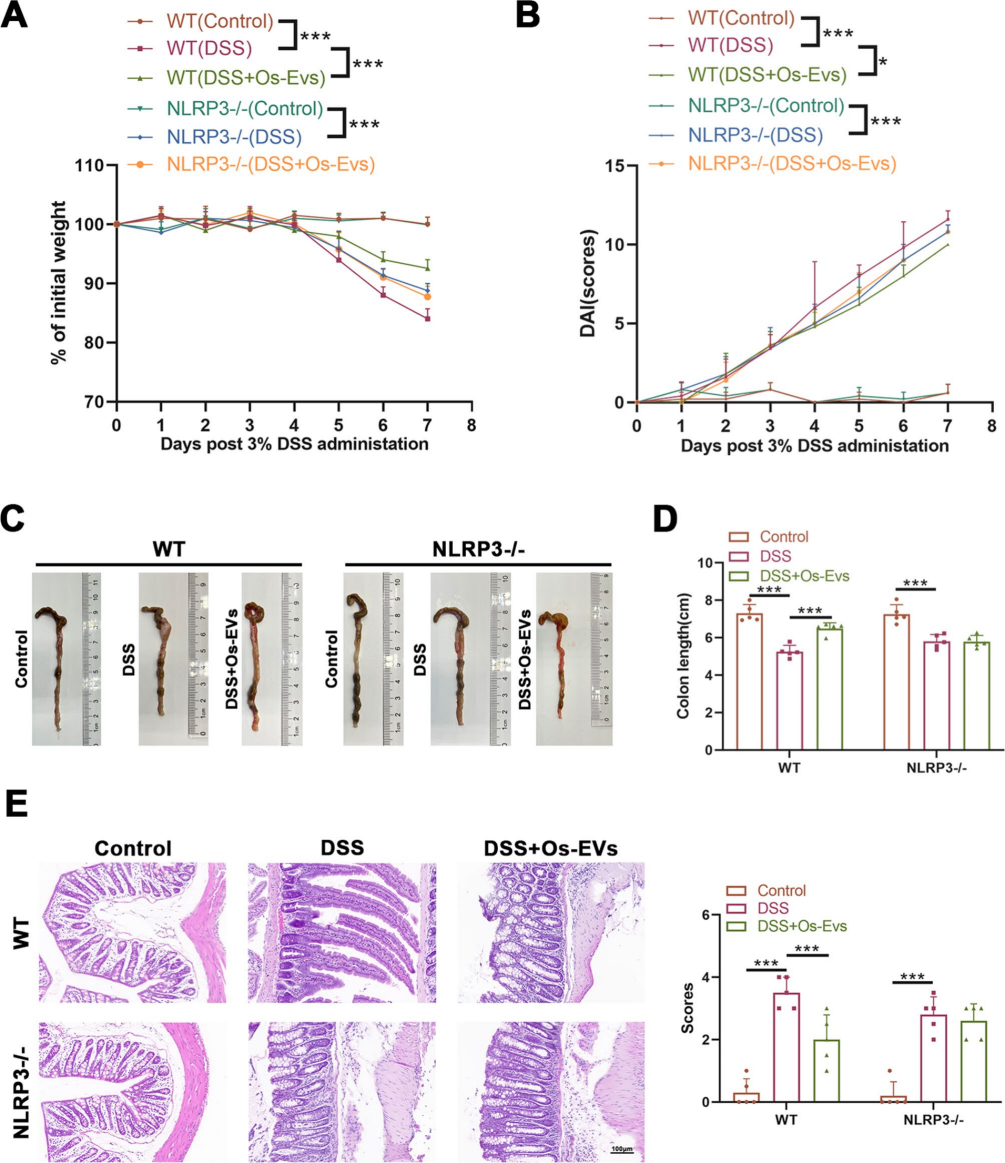
The protection of Os-EVs for DSS-induced IBD was diminished in NLRP3 deficient mice. **A**. Changes in body weight of each mouse were monitored every day (*n* = 5). **B**. DAI scores of each mouse were determined on the basis of the degree of weight loss, diarrhea, and hematochezia status after the administration of 3% DSS (*n* = 5). **C**. Representative images of the colon from both WT and NLRP3-/- mice of the control, DSS, and DSS + Os-EVs groups (*n* = 2). **D**. Length of the harvested colon from six groups (*n* = 5). **E**. Representative H&E-stained colonic slices among six groups (Scale bars represent 100 μm.). **F**. Histopathological scores were determined based on H&E-stained colonic slices among six groups (*n* = 5). Note: *means *P* < 0.05, ** means *P* < 0.01 and *** means *P* < 0.001

To explore whether Os-EVs play a role in attenuating IBD via NLRP3 inflammasome regulation, the functions of Os-EVs on DSS-induced IBD were further observed in NLRP3-/- mice. Similar to our previous experiments, the WT and NLRP3−/− mice were pretreated with or without Os-EVs during DSS induction. Consistently, WT mice pretreated with Os-EVs attenuated the IBD symptoms as revealed by less weight loss, DAI scores, and colon length shortening (Fig. 6A-D). However, there was no difference in body weight, scores, and colon length in the NLRP3−/− mice with or without pretreatment of Os-EVs (Fig. 6A-D). In conformity with the symptoms, compared WT mice in the DSS + Os-EVs group with the DSS group, the disappearance of crypts and inflammatory cell infiltration were less in the DSS + Os-EVs group under H&E staining observation (Fig. 6E). In the meantime, H&E staining did not show obvious alleviation in NLRP3−/− mice pretreated with Os-EVs compared with the DSS group (Fig. 6E). These results suggest that the role of Os-EVs in protecting against IBD depends on NLRP3.

### Knock out NLRP3 abolishes the role of Os-EVs in intestinal mucosal barrier, apoptosis, and inflammation of DSS-induced IBD mice

**Fig. 7:**
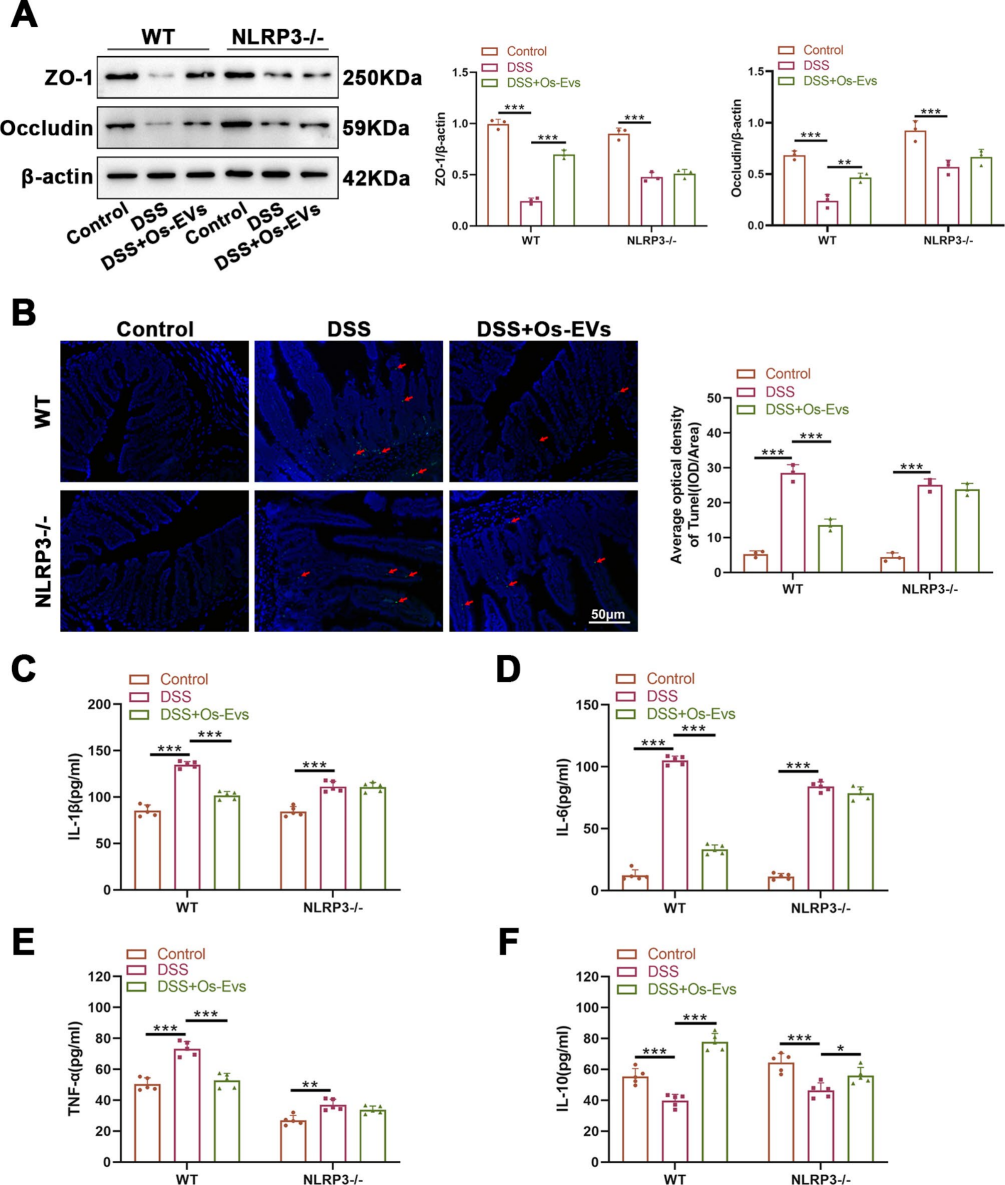
Knock out NLRP3 abolishes the role of Os-EVs in intestinal mucosal barrier, apoptosis, and inflammation of DSS-induced IBD mice. **A**. Protein levels of ZO-1 and Occludin in colon tissue analyzed by WB normalized to β-actin (*n* = 3). **B**. TUNEL staining for colonic section (*n* = 3). **C**-**F**. Serum IL-6, IL-1β, TNF-α and IL-10 levels were measured by ELISA from the mice (*n* = 5). Note: *means *P* < 0.05 and *** means *P* < 0.001

To further confirm our findings, the effects of Os-EVs on DSS-induced intestinal barrier dysfunction, apoptosis, and inflammation in NLRP3−/− mice were explored by conducting WB, TUNEL, and ELISA analyses. As expected, the protections of Os-EVs on DSS-induced IBD were observed in WT mice but not NLRP3−/− mice (Fig. 7). According to the results from WB analysis, under the DSS induction, the colonic expression of Occludin and ZO-1 of WT mice was lower than NLRP3-/-mice and Os-EVs improved the intestinal barrier dysfunction in WT mice but had no significant effect on NLRP3-/- mice (Fig. 7A). Similar tendencies were observed in both TUNEL staining (Fig. 7B) and ELISA analyses (Fig. 7C-F). These data corroborated that the relief of Os-EVs from DSS-induced IBD depended on the intervention of NLRP3 inflammasome.

## Discussion

Despite significant progress in treatments and clinical strategies for decreasing mortality rates from IBD in the last few decades, IBD still lead to life-threatening complications and increased mortality, especially in elderly patients (Follin-Arbelet et al. 2023). Changes in the gut microbiota is a critical biological indication as well as an important cause of IBD (Lavelle and Sokol 2020). In order to provide precise therapy for IBD, it is of importance to elucidating the specific mechanisms underlying intestinal diseases from the perspective of gut microbes and. In multiple research projects, the disordered gut microbiome was found in IBD individuals (Frank et al. 2007; Bartels et al. 2016; Vester-Andersen et al. 2019). More importantly, it was reported that the phenotypes of IBD can be transmitted to germ-free mice through the disordered intestinal microbiota (Jang et al. 2021). As mediators of intercellular communication, bacteria EVs have a capability of conveying bacterial constituents to host cells from great distances to trigger metabolic and immune responses (Shen et al. 2012). Due to the complicatedness of the intestinal microbiota, the functional roles of bacteria EVs in regulating the intestinal environment are diverse. In a previous study, EVs derived from *Fusobacterium nucleatum* can target the RIPK1-mediated epithelial cell death signal, thereby compromising the gut barrier (Liu et al. 2021). Additionally, an in vivo study based on mice revealed that *Akkermansia muciniphila*-derived EVs could attenuate DSS-induced colon length shortening, body weight loss, and so on (Kang et al. 2013).

As a commensal bacterium inhabiting the human intestinal tract, *O. splanchnicus* may beneficially activate intestinal health. Notably, Hiippala et al. demonstrated that outer membrane vesicles secreted by *O. splanchnicus* play immunoregulatory role by reducing IL-8 secretion and inducing IL-10 production in LPS-induced HT-29 cells (Hiippalaet al., 2020). In this study, the EVs secreted by probiotics (*O.splanchnicus*) alleviate the IBD symptoms, intestinal barrier dysfunction, apoptosis, and inflammation induced by DSS through regulating the NLRP3 inflammasome signaling.

It is acknowledged the important role of gut barrier function in IBD pathogenesis (McGuckin et al. 2009; Coskun 2014). Epithelial cells that bond with intercellular junction proteins play critical roles in maintaining intestinal barrier integrity (Ngo et al. 2022). Once intestinal epithelial cell injury, the integrity and tight junctions would respectively be impaired and loosened, causing a “leaky gut”. Accumulating evidence supports that the loss of barrier proteins is the beginning cause, which could result in immune disorder in IBD (Martini et al. 2017). Moreover, damage in the intestinal mucosal barrier function may further exacerbate the progression of IBD. Our data revealed that the expression of ZO-1 and Occludin in the colons could be elevated by pretreating with Os-EVs to some extent in DSS-induced IBD mice. Hence, the restoration role of Os-EVs in intestinal barrier function might partially illuminate its protective function on DSS-induced IBD in mice.

Generally, the persistent inflammation of IBD is considered as the customary feature and main mediator of its occurrence and progression. During IBD progression, the imbalance between pro- and anti-inflammatory cytokines impedes the resolution of inflammation and leads to tissue damage and disease persistence (Neurath 2014). Compelling evidence has demonstrated that increased cytotoxic components in the intestinal mucosa of IBD patients could lead to colonic destruction (Strober and Fuss 2011). Therefore, inflammatory pathways have served as lucrative targets to alleviate IBD. Our data displayed that the upregulation of proinflammatory cytokines (IL-1β, IL-6, and TNF-α) in the colon tissues and serum were reduced by pretreatment with Os-EVs whereas Os-EVs are capable of promoting the expression of IL-10 (a well-known anti-inflammatory cytokine) in DSS-induced mice, which further supported the protective effect of Os-EVs on IBD in mice.

The innate immune response to cell infection relies on receptors including Nod-like receptors (NLRs) (Schroder and Tschopp 2010); particularly, NLRP3 is one of the best-characterized receptors that is involved in multiple inflammatory diseases (Duewell et al. 2010). NLRP3 protein can bind to ASC and pro-caspase-1 to compose a cytoplasmic multimolecular platform, named the NLRP3 inflammasome. Once the NLRP3 inflammasome is activated, caspase-1 can be proteolytically activated by triggering cleavage, which causes subsequent secretion of IL-1β and IL-18 (Martinon et al. 2002). Notably, the increased release of IL-1β and IL-18 has been reported to cause the upregulation of IL-6 and TNF-α (Mahida et al. 1989). Besides, whether in the clinical samples of the ulcerated colon or the colon of mice with acute and chronic IBD, the overexpression of NLRP3 expression could be observed (Villani et al. 2009). Other research on intestinal inflammation also supports a pro-inflammatory contribution of NLRP3 to the pathology of IBD (Liu et al. 2013; Perera et al. 2018). Both neutralization of IL-18 and IL-1β blockage have been proven to reduce intestinal inflammation (Thomas et al. 1991; Ten Hove et al. 2001). In our study, the activation of NLRP3 inflammasome was observed in DSS-induced mice, whereas Os-EVs pretreatment could partly block the activation of NLRP3 inflammasome. To explore whether Os-EVs play a role in attenuating IBD via NLRP3 inflammasome, the effects of Os-EVs on DSS-induced IBD were further observed in NLRP3 knockout mice. Studies have demonstrated that mice with NLRP3 knockout, when exposed to DSS, exhibit lower levels of proinflammatory cytokines in colonic tissue and manifest less severe colitis compared to their WT counterparts (Gao et al. 2021; Bauer et al. 2010). Similarly, our study showed that NLRP3 knockout mice developed less severe DSS-induced IBD compared to the WT, supporting the critical role of NLRP3 in IBD development. In addition, Os-EVs-mediated amelioration in DSS-induced IBD was not further enhanced in NLPR3 knockout mice, which suggested that Os-EVs exert a function of protecting against DSS-induced IBD via the suppression of the NLRP3 signaling pathways. Nevertheless, the specific molecular mechanism of how Os-EVs suppress the NLRP3 inflammasome remains unclear, which is required to be explored in future studies.

Previous studies demonstrated that the therapeutic role of some interventions in IBD may partly rely on their function of remodeling the intestinal microbiota (Tao et al. 2024; Tong et al. 2021). For example, milk-derived EVs have been proven to alleviate ulcerative colitis by increasing the abundance of some beneficial gut microbes, such as *Akkermansia* (Tong et al. 2021). A more recent study demonstrated that *Limosilactobacillus mucosae* intervention could attenuate diarrheal disease symptoms which simultaneously increased the fecal abundance of probiotic bacterium such as *Lactobacillus spp.*, in mice (Tao et al. 2024). Given the critical role of gut microbiota in IBD, whether Os-EVs influence the overall gut microbiota composition and function remains to be explored.

Taken together, our study, for the first time, identified the therapeutic effects of Os-EVs on IBD and preliminarily uncovered the underlying mechanism. However, there are several limitations in the present study. Although our findings indicated the function of protecting against DSS-induced IBD in mice, the dose-response relationship as well as the long-term effects of Os-EVs on IBD were not evaluated. Further research is needed to assess the chronic safety and efficacy of Os-EVs on IBD before it can be considered for clinical application. Besides, further studies need to identify the specific molecular cargo contained within Os-EVs and to clarify a more detailed mechanism behind the role of Os-EVs in IBD.

## Conclusion

In summary, the present research demonstrated that pretreatment of Os-EVs alleviated DSS-induced IBD in mice through regulating the intestinal barrier and inflammation, paving the way for a promising strategy in IBD treatment. Further studies are needed to uncover the exact mechanisms and identify the specific components of Os-EVs that mediate the intestinal protection effect on IBD.

## Data Availability

No datasets were generated or analysed during the current study.

## Abbreviations

IBD: Inflammatory bowel disease
EVs: Extracellular vesicles
*O.splanchnicus*: *Odoribacter splanchnicus*
Os-EVs: Extracellular vesicles derived from *Odoribacter splanchnicus*
DSS: Dextran sulfate sodium
IL: Interleukin
SPF: Specified pathogen-free
WT: Wild-type
PBS: Phosphate-buffered saline
TEM: Transmission electron microscope
NTA: Nanoparticle tracking analysis
H&E: Hematoxylin & Eosin
IF: Immunofluorescent
ZO-1: Zonula occludens-1
DAPI: 4,6′-Diamidino-2-phenylindole
ASC: Apoptosis-associated speck-like protein containing a CARD
TUNEL: Terminal deoxynucleotidyl transferase dUTP nick end labeling
IHC: Immunohistochemical

## References

[CR1] Bartels LE, Jepsen P, Christensen LA, Gerdes LU, Vilstrup H, Dahlerup JF. Diagnosis of Helicobacter Pylori Infection is Associated with Lower Prevalence and subsequent incidence of Crohn’s Disease. J Crohns Colitis. 2016;10(4):443–8. 26674958 10.1093/ecco-jcc/jjv229 PMC4946761

[CR2] Bauer C, Duewell P, Mayer C, Lehr HA, Fitzgerald KA, Dauer M, et al. Colitis induced in mice with dextran sulfate sodium (DSS) is mediated by the NLRP3 inflammasome. Gut. 2010;59(9):1192–9. 20442201 10.1136/gut.2009.197822

[CR3] Champagne-Jorgensen K, Jose TA, Stanisz AM, Mian MF, Hynes AP, Bienenstock J. Bacterial membrane vesicles and phages in blood after consumption of lacticaseibacillus rhamnosus JB-1. Gut Microbes. 2021;13(1):1993583. 34747333 10.1080/19490976.2021.1993583 PMC8583084

[CR4] Coskun M. Intestinal epithelium in inflammatory bowel disease. Front Med (Lausanne). 2014;1:24. 25593900 10.3389/fmed.2014.00024 PMC4292184

[CR5] de Souza HS, Fiocchi C. Immunopathogenesis of IBD: current state of the art. Nat Rev Gastroenterol Hepatol. 2016;13(1):13–27. 26627550 10.1038/nrgastro.2015.186

[CR6] Duewell P, Kono H, Rayner KJ, Sirois CM, Vladimer G, Bauernfeind FG, et al. NLRP3 inflammasomes are required for atherogenesis and activated by cholesterol crystals. Nature. 2010;464(7293):1357–61. 20428172 10.1038/nature08938 PMC2946640

[CR7] Ekstedt N, Jamioł-Milc D, Pieczyńska J. Importance of gut microbiota in patients with inflammatory bowel disease. Nutrients. 2024;16(13). 10.3390/nu16132092 PMC11242987 38999840

[CR8] Follin-Arbelet B, Cvancarova Småstuen M, Hovde Ø, Jelsness-Jørgensen LP, Moum B. Mortality in patients with inflammatory bowel disease: results from 30 years of follow-up in a Norwegian inception cohort (the IBSEN study). J Crohns Colitis. 2023;17(4):497–503. 36239614 10.1093/ecco-jcc/jjac156 PMC10115228

[CR9] Frank DN, St Amand AL, Feldman RA, Boedeker EC, Harpaz N, Pace NR. Molecular-phylogenetic characterization of microbial community imbalances in human inflammatory bowel diseases. Proc Natl Acad Sci U S A. 2007;104(34):13780–5. 17699621 10.1073/pnas.0706625104 PMC1959459

[CR10] Gao H, Cao M, Yao Y, Hu W, Sun H, Zhang Y, et al. Dysregulated microbiota-driven gasdermin D activation promotes Colitis Development by mediating IL-18 release. Front Immunol. 2021;12:750841. 34721422 10.3389/fimmu.2021.750841 PMC8551709

[CR11] Göker M, Gronow S, Zeytun A, Nolan M, Lucas S, Lapidus A, et al. Complete genome sequence of Odoribacter splanchnicus type strain (1651/6). Stand Genomic Sci. 2011;4(2):200–9. 21677857 10.4056/sigs.1714269 PMC3111987

[CR58] Hiippala K, Barreto G, Burrello C, Diaz-Basabe A, Suutarinen M, Kainulainen V, et al. Novel Odoribacter splanchnicus Strain and Its Outer Membrane Vesicles Exert Immunoregulatory Effects in vitro. Front Microbiol. 2020;11:575455. 10.3389/fmicb.2020.575455 PMC7689251 33281770

[CR12] Jang HM, Kim JK, Joo MK, Shin YJ, Lee CK, Kim HJ, et al. Transplantation of fecal microbiota from patients with inflammatory bowel disease and depression alters immune response and behavior in recipient mice. Sci Rep. 2021;11(1):20406. 34650107 10.1038/s41598-021-00088-x PMC8516877

[CR13] Kang CS, Ban M, Choi EJ, Moon HG, Jeon JS, Kim DK, et al. Extracellular vesicles derived from gut microbiota, especially Akkermansia muciniphila, protect the progression of dextran sulfate sodium-induced colitis. PLoS ONE. 2013;8(10):e76520. 24204633 10.1371/journal.pone.0076520 PMC3811976

[CR14] Kim JH, Jeun EJ, Hong CP, Kim SH, Jang MS, Lee EJ, et al. Extracellular vesicle-derived protein from Bifidobacterium longum alleviates food allergy through mast cell suppression. J Allergy Clin Immunol. 2016;137(2):507–. – 16.e8. 26433560 10.1016/j.jaci.2015.08.016

[CR15] Kumar MA, Baba SK, Sadida HQ, Marzooqi SA, Jerobin J, Altemani FH, et al. Extracellular vesicles as tools and targets in therapy for diseases. Signal Transduct Target Ther. 2024;9(1):27. 38311623 10.1038/s41392-024-01735-1 PMC10838959

[CR16] Kuo WT, Zuo L, Odenwald MA, Madha S, Singh G, Gurniak CB, et al. The tight Junction protein ZO-1 is dispensable for barrier function but critical for effective mucosal repair. Gastroenterology. 2021;161(6):1924–39. 34478742 10.1053/j.gastro.2021.08.047 PMC8605999

[CR17] Landy J, Ronde E, English N, Clark SK, Hart AL, Knight SC, et al. Tight junctions in inflammatory bowel diseases and inflammatory bowel disease associated colorectal cancer. World J Gastroenterol. 2016;22(11):3117–26. 27003989 10.3748/wjg.v22.i11.3117 PMC4789987

[CR18] Lavelle A, Sokol H. Gut microbiota-derived metabolites as key actors in inflammatory bowel disease. Nat Rev Gastroenterol Hepatol. 2020;17(4):223–37. 32076145 10.1038/s41575-019-0258-z

[CR19] Lees CW, Barrett JC, Parkes M, Satsangi J. New IBD genetics: common pathways with other diseases. Gut. 2011;60(12):1739–53. 21300624 10.1136/gut.2009.199679

[CR20] Lemon KP, Armitage GC, Relman DA, Fischbach MA. Microbiota-targeted therapies: an ecological perspective. Sci Transl Med. 2012;4(137):137rv5. 22674555 10.1126/scitranslmed.3004183 PMC5725196

[CR21] Lewis JD, Chen EZ, Baldassano RN, Otley AR, Griffiths AM, Lee D, et al. Inflammation, antibiotics, and Diet as Environmental stressors of the gut Microbiome in Pediatric Crohn’s Disease. Cell Host Microbe. 2015;18(4):489–500. 26468751 10.1016/j.chom.2015.09.008 PMC4633303

[CR22] Li W, Sun Y, Dai L, Chen H, Yi B, Niu J, et al. Ecological and network analyses identify four microbial species with potential significance for the diagnosis/treatment of ulcerative colitis (UC). BMC Microbiol. 2021;21(1):138. 33947329 10.1186/s12866-021-02201-6 PMC8097971

[CR23] Lima SF, Gogokhia L, Viladomiu M, Chou L, Putzel G, Jin WB, et al. Transferable immunoglobulin A-Coated Odoribacter splanchnicus in responders to fecal microbiota transplantation for ulcerative colitis limits colonic inflammation. Gastroenterology. 2022;162(1):166–78. 34606847 10.1053/j.gastro.2021.09.061 PMC8678328

[CR25] Liu TC, Stappenbeck TS. Genetics and Pathogenesis of Inflammatory Bowel Disease. Annu Rev Pathol. 2016;11:127–48. 26907531 10.1146/annurev-pathol-012615-044152 PMC4961083

[CR26] Liu W, Guo W, Wu J, Luo Q, Tao F, Gu Y, et al. A novel benzo[d]imidazole derivate prevents the development of dextran sulfate sodium-induced murine experimental colitis via inhibition of NLRP3 inflammasome. Biochem Pharmacol. 2013;85(10):1504–12. 23506741 10.1016/j.bcp.2013.03.008

[CR27] Liu Y, Defourny KAY, Smid EJ, Abee T. Gram-positive bacterial extracellular vesicles and their impact on Health and Disease. Front Microbiol. 2018;9:1502. 30038605 10.3389/fmicb.2018.01502 PMC6046439

[CR24] Liu L, Liang L, Yang C, Zhou Y, Chen Y. Extracellular vesicles of Fusobacterium nucleatum compromise intestinal barrier through targeting RIPK1-mediated cell death pathway. Gut Microbes. 2021;13(1):1–20. 33769187 10.1080/19490976.2021.1902718 PMC8007154

[CR28] Mahida YR, Wu K, Jewell DP. Enhanced production of interleukin 1-beta by mononuclear cells isolated from mucosa with active ulcerative colitis of Crohn’s disease. Gut. 1989;30(6):835–8. 2787769 10.1136/gut.30.6.835 PMC1434123

[CR29] Mai CT, Wu MM, Wang CL, Su ZR, Cheng YY, Zhang XJ. Palmatine attenuated dextran sulfate sodium (DSS)-induced colitis via promoting mitophagy-mediated NLRP3 inflammasome inactivation. Mol Immunol. 2019;105:76–85. 30496979 10.1016/j.molimm.2018.10.015

[CR30] Martini E, Krug SM, Siegmund B, Neurath MF, Becker C. Mend your fences: the epithelial barrier and its Relationship with Mucosal Immunity in Inflammatory Bowel Disease. Cell Mol Gastroenterol Hepatol. 2017;4(1):33–46. 28560287 10.1016/j.jcmgh.2017.03.007 PMC5439240

[CR31] Martinon F, Burns K, Tschopp J. The inflammasome: a molecular platform triggering activation of inflammatory caspases and processing of proIL-beta. Mol Cell. 2002;10(2):417–26. 12191486 10.1016/s1097-2765(02)00599-3

[CR32] McGuckin MA, Eri R, Simms LA, Florin TH, Radford-Smith G. Intestinal barrier dysfunction in inflammatory bowel diseases. Inflamm Bowel Dis. 2009;15(1):100–13. 18623167 10.1002/ibd.20539

[CR33] Morgan XC, Tickle TL, Sokol H, Gevers D, Devaney KL, Ward DV, et al. Dysfunction of the intestinal microbiome in inflammatory bowel disease and treatment. Genome Biol. 2012;13(9):R79. 23013615 10.1186/gb-2012-13-9-r79 PMC3506950

[CR34] Neurath MF. Cytokines in inflammatory bowel disease. Nat Rev Immunol. 2014;14(5):329–42. 24751956 10.1038/nri3661

[CR35] Ngo PA, Neurath MF, López-Posadas R. Impact of epithelial cell shedding on intestinal homeostasis. Int J Mol Sci 2022;23(8). 10.3390/ijms23084160 PMC9027054 35456978

[CR36] Ni J, Wu GD, Albenberg L, Tomov VT. Gut microbiota and IBD: causation or correlation? Nat Rev Gastroenterol Hepatol. 2017;14(10):573–84. 28743984 10.1038/nrgastro.2017.88 PMC5880536

[CR37] Parada Venegas D, De la Fuente MK, Landskron G, González MJ, Quera R, Dijkstra G, et al. Short chain fatty acids (SCFAs)-Mediated gut epithelial and Immune Regulation and its relevance for inflammatory Bowel diseases. Front Immunol. 2019;10:277. 30915065 10.3389/fimmu.2019.00277 PMC6421268

[CR38] Perera AP, Fernando R, Shinde T, Gundamaraju R, Southam B, Sohal SS, et al. MCC950, a specific small molecule inhibitor of NLRP3 inflammasome attenuates colonic inflammation in spontaneous colitis mice. Sci Rep. 2018;8(1):8618. 29872077 10.1038/s41598-018-26775-w PMC5988655

[CR39] Sann H, Erichsen J, Hessmann M, Pahl A, Hoffmeyer A. Efficacy of drugs used in the treatment of IBD and combinations thereof in acute DSS-induced colitis in mice. Life Sci. 2013;92(12):708–18. 23399699 10.1016/j.lfs.2013.01.028

[CR40] Sartor RB, Wu GD. Roles for intestinal Bacteria, viruses, and Fungi in Pathogenesis of Inflammatory Bowel diseases and therapeutic approaches. Gastroenterology. 2017;152(2):327–e394. 27769810 10.1053/j.gastro.2016.10.012 PMC5511756

[CR41] Schieber AM, Lee YM, Chang MW, Leblanc M, Collins B, Downes M, et al. Disease tolerance mediated by microbiome E. Coli involves inflammasome and IGF-1 signaling. Science. 2015;350(6260):558–63. 26516283 10.1126/science.aac6468 PMC4732872

[CR42] Schroder K, Tschopp J. The inflammasomes. Cell. 2010;140(6):821–32. 20303873 10.1016/j.cell.2010.01.040

[CR44] Shen Y, Giardino Torchia ML, Lawson GW, Karp CL, Ashwell JD, Mazmanian SK. Outer membrane vesicles of a human commensal mediate immune regulation and disease protection. Cell Host Microbe. 2012;12(4):509–20. 22999859 10.1016/j.chom.2012.08.004 PMC3895402

[CR43] Shen Q, Huang Z, Yao J, Jin Y. Extracellular vesicles-mediated interaction within intestinal microenvironment in inflammatory bowel disease. J Adv Res. 2022;37:221–33. 35499059 10.1016/j.jare.2021.07.002 PMC9039646

[CR45] Smillie CS, Sauk J, Gevers D, Friedman J, Sung J, Youngster I, et al. Strain Tracking reveals the determinants of bacterial engraftment in the human gut following fecal microbiota transplantation. Cell Host Microbe. 2018;23(2):229–e405. 29447696 10.1016/j.chom.2018.01.003 PMC8318347

[CR46] Strober W, Fuss IJ. Proinflammatory cytokines in the pathogenesis of inflammatory bowel diseases. Gastroenterology. 2011;140(6):1756–67. 21530742 10.1053/j.gastro.2011.02.016 PMC3773507

[CR47] Tao S, Fan J, Li J, Wu Z, Yao Y, Wang Z et al. Extracellular vesicles derived from Lactobacillus johnsonii promote gut barrier homeostasis by enhancing M2 macrophage polarization. J Adv Res. 2024.10.1016/j.jare.2024.03.01138508446

[CR48] Ten Hove T, Corbaz A, Amitai H, Aloni S, Belzer I, Graber P, et al. Blockade of endogenous IL-18 ameliorates TNBS-induced colitis by decreasing local TNF-alpha production in mice. Gastroenterology. 2001;121(6):1372–9. 11729116 10.1053/gast.2001.29579

[CR49] Thomas TK, Will PC, Srivastava A, Wilson CL, Harbison M, Little J, et al. Evaluation of an interleukin-1 receptor antagonist in the rat acetic acid-induced colitis model. Agents Actions. 1991;34(1–2):187–90. 1838896 10.1007/BF01993274

[CR50] Tong L, Hao H, Zhang Z, Lv Y, Liang X, Liu Q, et al. Milk-derived extracellular vesicles alleviate ulcerative colitis by regulating the gut immunity and reshaping the gut microbiota. Theranostics. 2021;11(17):8570–86. 34373759 10.7150/thno.62046 PMC8344018

[CR51] Vester-Andersen MK, Mirsepasi-Lauridsen HC, Prosberg MV, Mortensen CO, Träger C, Skovsen K, et al. Increased abundance of proteobacteria in aggressive Crohn’s disease seven years after diagnosis. Sci Rep. 2019;9(1):13473. 31530835 10.1038/s41598-019-49833-3 PMC6748953

[CR52] Villani AC, Lemire M, Fortin G, Louis E, Silverberg MS, Collette C, et al. Common variants in the NLRP3 region contribute to Crohn’s disease susceptibility. Nat Genet. 2009;41(1):71–6. 19098911 10.1038/ng285 PMC2728932

[CR54] Wang Y, Gao X, Ghozlane A, Hu H, Li X, Xiao Y, et al. Characteristics of Faecal Microbiota in Paediatric Crohn’s Disease and their dynamic changes during Infliximab Therapy. J Crohns Colitis. 2018;12(3):337–46. 29194468 10.1093/ecco-jcc/jjx153

[CR53] Wang X, Lin S, Wang L, Cao Z, Zhang M, Zhang Y, et al. Versatility of bacterial outer membrane vesicles in regulating intestinal homeostasis. Sci Adv. 2023;9(11):eade5079. 36921043 10.1126/sciadv.ade5079 PMC10017049

[CR55] Welsh JA, Goberdhan DCI, O’Driscoll L, Buzas EI, Blenkiron C, Bussolati B, et al. Minimal information for studies of extracellular vesicles (MISEV2023): from basic to advanced approaches. J Extracell Vesicles. 2024;13(2):e12404. 38326288 10.1002/jev2.12404 PMC10850029

[CR56] Wirtz S, Popp V, Kindermann M, Gerlach K, Weigmann B, Fichtner-Feigl S, et al. Chemically induced mouse models of acute and chronic intestinal inflammation. Nat Protoc. 2017;12(7):1295–309. 28569761 10.1038/nprot.2017.044

[CR57] Xu J, Xu H, Guo X, Wang J, Zhu M, Zhao H et al. IDDF2023-ABS-0100 Odoribacter. Splanchnicus alleviate colitis by regulating neutrophil extracellular traps formation. 2023;72:A79–80.

